# Health Resorts

**Published:** 1889-07

**Authors:** 


### HALL'S JOURNAL OF HEALTH

TRUTH DEMANDS NO SACRIFICE: ERROR CAN MAKE NONE.

VOL. 36. JULY, 1889. No. 7.

### HEALTH RESORTS.

This is a season when the impaired energies of mind and body are accustomed to seek recuperation, and it becomes a serious question where to go. It is generally conceded that persons who habitually dwell upon, or contiguous to the sea, find the greater benefit in the highlands, and the converse proposition is true in respect to those whose homes are among the mountains. The States of New York, New Jersey, Pennsylvania and New England are particularly favored in the variety of their seashore and highland resorts. There are ocean beaches, so easy of access and so popular that even the corruptions of wealth and fashion cannot spoil them. Then there are numberless quiet villages nestled among the hills, and leafy, secluded bays, where the health seeker is not expected to sacrifice the comforts of life to the flummery of the idle and extravagant. It is in these less known, but more desirable resorts that the more solid enjoyment, such as conduces to good health by right living, is to be found.

There is no more desirable inland resort than the mountain region of New Hampshire, made so familiar to us by the admirable work of the late Thomas Star King. Nor are the seashore resorts of this State and the far eastern ones of Maine, surpassed by any others of which we have any knowledge. Who that has visited the beautiful Lake Winnipesaukee and toyed with its waters in the warm July days, or the wide sandy reach of Hampton Beach, but longs to repeat his visit when the season returns with its holiday frolics and fascinations? What more delightful than a sail among the three hundred islands of the lake, or to one so inclined, a fishing party where the pearch, the pickerel and bass abound, but not the trout; they seek the cool sources of the lake at unknown depths beneath its silvery crest in summer time, to reappear when the chill winter has laid the crystal wave beneath its pall. We have for many a year held this lovely region sacred to our trust of life and health and aims for the accomplishment of good, for only by the enjoyment of good health are we able to do good work.

"There is a pleasure in the pathless woods,
There is a rapture on the lonely shore,
There is society, where none intrudes,
By the deep sea, and music in its roar."

And there is nothing more healthful than innocent pleasures, which find an ever welcome response in all true loving hearts. The man who has outlived them is to be pitied.

